# A report of nine cases and review of the literature of infertile men carrying balanced translocations involving chromosome 5

**DOI:** 10.1186/s13039-018-0360-x

**Published:** 2018-01-25

**Authors:** Hong-Guo Zhang, Rui-Xue Wang, Yuan Pan, Han Zhang, Lei-Lei Li, Hai-Bo Zhu, Rui-Zhi Liu

**Affiliations:** Center for Reproductive Medicine and Center for Prenatal Diagnosis, First Hospital, Jilin University, 71 Xinmin Street, Chaoyang District, Changchun, Jilin Province 130021 China

**Keywords:** Male infertility, Chromosome 5, Balanced translocation, Breakpoint, Genetic counseling

## Abstract

**Background:**

Balanced translocations may cause the loss of genetic material at the breakpoints and may result in failure of spermatogenesis. However, carriers of reciprocal translocation may naturally conceive. Genetic counseling of male carriers of translocations remains challenging. This study explores the clinical features of carriers of chromosome 5 translocations, enabling informed genetic counseling of these patients.

**Results:**

Of 82 translocation carriers, 9 (11%) were carriers of a chromosome 5 translocation. One case had azoospermia, while three cases had experienced recurrent spontaneous abortions, two cases had each experienced stillbirth, and three cases produced a phenotypically normal child confirmed by amniocentesis. A literature review identified 106 patients who carried chromosome 5 translocations. The most common chromosome 5 translocation was t(4,5), observed in 13 patients. Breakpoint at 5p15 was observed in 11 patients. All breakpoints at chromosome 5 were associated with gestational infertility.

**Conclusion:**

In genetic counseling, physicians should consider chromosome 5 and its breakpoints. Carriers of chromosome 5 translocations may continue with natural conception or use assisted reproductive technologies, such as preimplantation genetic diagnosis.

## Background

Known chromosomal alterations play a major role in perturbing male fertility [1]. Reciprocal chromosomal translocations are the most common structural rearrangement, with an incidence in infertile males up to ten times higher than in fertile men [2]. Balanced chromosomal translocations may cause the loss of genetic material at breakpoints and may result in failure of spermatogenesis [3]. Individuals affected by such translocations exhibit reproductive problems such as infertility, recurrent pregnancy loss, and malformed offspring [4, 5]. These effects are related to the specific chromosomes and breakpoints involved in the translocation [6, 7]. Some breakpoints can disrupt the structure of an important gene, leading to spermatogenic or maturation disorders, and male infertility [5]. Important genes associated with male infertility are located on chromosome 5. For example, *Camk4* (encoding Ca^2+^/calmodulin-dependent protein kinase IV) is located on chromosome 5q22.1, and is expressed in spermatids and associated with chromatin and nuclear matrix [8]. Disrupted *CAMK4* expression may be associated with human male infertility [8]. In addition, the *Spink13* gene (encoding serine protease inhibitor, Kazal-type 13), mapped on chromosome 5 at 5q32, was reported to be associated with sperm maturation [9]. The breakpoint of 5p13 was shown to be related to impaired spermatogenesis [10].

However, genetic counseling of male carriers of chromosomal translocations remains challenging. Preimplantation genetic diagnosis (PGD) is recommended for those exhibiting a balanced translocation. Microdissection testicular sperm extraction and in vitro fertilization accompanied by PGD increases the chance of these carriers fathering a healthy child [11, 12]. Clinical characteristics, including spontaneous abortion, do not differ between those couples who accept and those who decline PGD [13]. A systematic review showed there was insufficient evidence that PGD improves the live birth rate in couples with repeated miscarriage and carrying a structural chromosome abnormality [14]. In addition, the live birth rate in patients refused PGD and choosing to conceive naturally was reported to be 37–63% for the first pregnancy, and then a cumulative rate of 65–83% [4]. Natural pregnancy success rates for couples in which the male carries a chromosomal translocation ranged from 30% to 70% [15]. This suggests that continued attempts to conceive naturally are a viable option for successful pregnancy, however, the relationship between clinical features and chromosome structural abnormality warrants further study.

The present study was established to explore the clinical features and translocation breakpoints in carriers of balanced translocations involving chromosome 5. This paper also highlights the importance of genetic counseling for infertile men.

## Methods

### Patients

Between July 2010 and December 2016, 82 male carriers of chromosomal translocations who were experiencing infertility, or receiving counseling, were recruited from the outpatient’s department at the Center for Reproductive Medicine, the First Hospital of Jilin University, Changchun, China. All patients underwent a thorough physical examination and semen analysis, and were required to complete a detailed questionnaire regarding their smoking habits, marital status, medical history, and working conditions. The study protocol was approved by the Ethics Committee of the First Hospital of Jilin University, and written informed consent was obtained from all participants.

### Semen analysis

Semen analysis was performed according to procedures recommended by the World Health Organization guidelines. If no sperm was found, sperm was analyzed by sedimenting semen samples through centrifugation. Patients with oligozoospermia were diagnosed as a sperm count less than 15 × 10^6^/ml in their last three semen samples (taken at intervals of 1–3 weeks). Azoospermia and oligozoospermia were defined as previously described [5]. All analyses were performed at the same laboratory, and all data were accessed from medical records.

### Cytogenetic analysis

Cytogenetic analysis was carried out on all patients. Peripheral blood (0.5 mL) was collected in sterile tubes containing 30 U/mL heparin. Lymphocytes were then cultured in appropriate culture medium (Yishengjun; Guangzhou Baidi Biotech, Guangzhou, China) for 72 h, and subsequently treated with 20 μg/mL colcemid for 1 h. G-banding of metaphase chromosomes and karyotype analysis were performed using previously described methods [5]. Twenty metaphases were counted and 6 karyotypes were analyzed per patient. Karyotype nomenclature was described in accordance of ISCN2009. The resolution level of chromosome analysis was 400–550 band levels.

### Analysis of the identified translocation breakpoints

A search of translocations identified in chromosome 5 from infertile males was performed using PubMed. The keywords “chromosome / translocation / sperm” and “chromosome / translocation / abortion” were used for the PubMed search. The relationships of translocation breakpoints with male infertility and recurrent pregnancy loss were analyzed. Such searches were performed for a total of 106 carriers of chromosomal 5 translocations. This study included the cases of reciprocal chromosomal translocations involving chromosome 5 in reported papers, and excluded cases without breakpoints, females, newborns, and bone marrow detection involving chromosome 5.

## Results

**Fig. 1:**
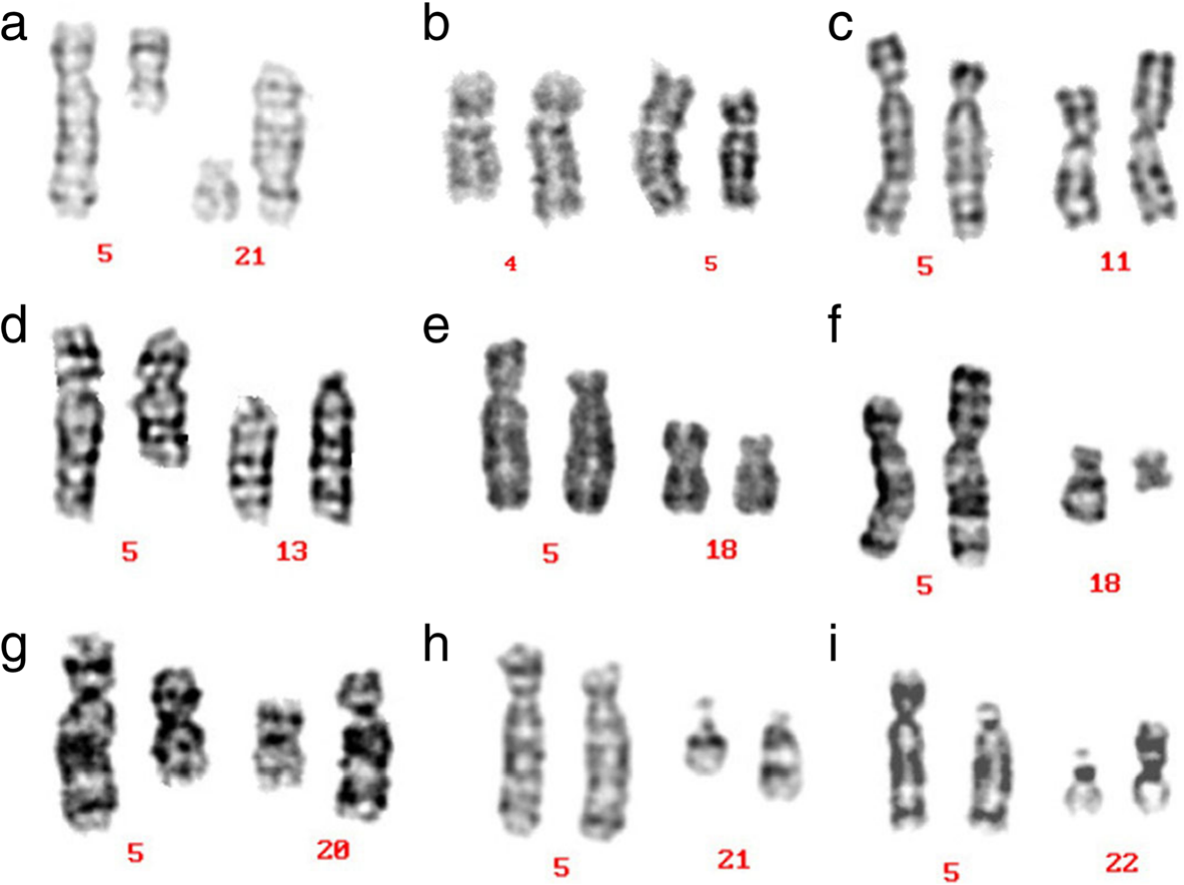
G-banding karyotype of the nine cases identified as possessing chromosome 5 translocations. **a**: t(5;21), **b**: t(4;5), **c**: t(5;11), **d**:t(5;13), **e**: t(5;18)(p13;p11), **f**: t(5;18)(p15;q11.2), **g**: t(5; 20), **h**: t(5;21), **i**: t(5;22)

A total of 82 translocation carriers were detected in this study. Of these, nine (11%) were carriers of a chromosome 5 translocation, in which other chromosome abnormality had been excluded. Karyotype results and G-banding karyotypes from these nine patients are respectively summarized in Table 1 and Fig. 1. One case had azoospermia (pregestational infertility), while eight cases had normal semen. For the former, no AZF gene deletion was found. Of the later eight cases, it was evident that their partners were able to conceive, but had a tendency to miscarry (gestational infertility): three cases had experienced recurrent spontaneous abortions, two cases each experienced stillbirths, and three cases produced a phenotypically normal child confirmed by amniocentesis. For the other 73 cases of translocations, we will describe or have published in another study.

**Table 1:** Karyotypes of chromosome 5 translocation carriers and their clinical features

Infertility causes	Clinical findings	Karyotype	Giemsa banding
Pregestational infertility	Azoospermia	46,XY,t(5;21)(q13;p12)	Figure 1a
Gestational infertility	Normal sperm density; a history of miscarriage, or normal fertility	46,XY,t(4;5)(q31;p15)	Figure 1b
		46,XY,t(5;11)(p14;p15)	Figure 1c
		46,XY,t(5;13)(q13;q12)	Figure 1d
		46,XY,t(5;18)(p13;p11)	Figure 1e
		46,XY,t(5;18)(p15;q11.2)	Figure 1f
		46,XY,t(5;20)(q13;q12)	Figure 1g
		46,XY,t(5;21)(p13;q22)	Figure 1h
		46,XY,t(5;22)(p11;p11)	Figure 1i

A literature review was also performed, from which karyotype results, clinical manifestations, and breakpoints on chromosome 5 were collected, as shown in Table 2. A total of 106 karyotypes included chromosome 5 translocations. The most common translocation was t(4;5), observed in 13 patients, followed t(5;8) (*N* = 11). Chromosomes 4(*N* = 13), 8(N = 11), 2,3,7,13(*N* = 7), 1,9,10,12(*N* = 6), 6, 18(*N* = 5), 15,20(*N* = 4),14,16,17(*N* = 3) and 11,19, X (N = 1) were respectively involved in the balanced translocation with chromosome 5.

**Table 2:** Breakpoints in chromosome 5 translocation carriers and clinical features

Case	Karyotype	Breakpoints	Clinical findings	Reference
1	t(1;5)	1p32;5q31	Severe oligoasthenoteratozoospermia	Peschka et al., 1999 [27]
2	t(1;5)	1p31.1;5q33.3	Normozoospermia	Brugnon et al., 2006 [28]
3	t(1;5)	1p22;5q11	Malformed/stillborn children	Meza-Espinoza et al., 2008 [29]
4	t(1;5)	1q36.1;5q31	2 miscarriage, PGD and 2 term delivery	Ikuma et al., 2015 [4]
5	t(1;5)	1q41;5q33	Miscarriage and PGD	Kyu Lim et al., 2004 [30]
6	t(1;5)	1qter;5p14	Recurrent miscarriage	Goud et al., 2009 [31]
7	t(2;5)	2p25;5p12	Teratozoospermia, Habitual abortions	Vegetti et al., 2000 [32]
8	t(2;5)	2p21;5p15	Recurrent spontaneous abortion	Gada Saxena et al., 2012 [33]
9	t(2;5)	2p13;5p15	Recurrent fetal wastage	Fryns et al., 1998 [34]
10	t(2;5)	2p11;5q15	Abortion	Templado et al., 1988 [35]
11	t(2;5)	2p11;5q31	Recurrent abortion	Portnoï et al., 1988 [36]
12	t(2;5)	2q12;5q35.3	Recurrent pregnancy loss	Kochhar et al., 2013 [22]
13	t(2;5)	2q13.1;5q35.1	6 miscarriage, PGD and 1 term delivery	Ikuma et al., 2015 [4]
14	t(3;5)	3p27;5p14	4 fetal losses	Adamoli et al., 1986 [37]
15	t(3;5)	3q13;5q35	Repeated abortions	Venkateshwari et al., 2011 [21]
16	t(3;5)	3q26.2;5p15.1	Miscarriage	Sugiura-Ogasawara et al., 2008 [38]
17	t(3;5)	3q27;5p15	Normospermic, A boy 46,XY,t(3;5)pat	Vozdova et al., 2013 [11]
18	t(3;5)	3q28;5p13	Recurrent spontaneous abortion	Gada Saxena et al., 2012 [33]
19	t(3;5)	3q29;5q13	Multiple abortions	Castle et al., 1988 [39]
20	t(3;5)	3q29;5q33.2	PGD	Findikli et al., 2003 [40]
21	t(4;5)	4p15.2;5p12	Normozoospermia	Wiland et al., 2007 [41]
22	t(4;5)	4p15;5q12	Oligozoospermia	Perrin et al., 2010 [42]
23	t(4;5)	4p14;5q13.1	recurrent miscarriage	Pundir et al., 2016 [43]
24	t(4;5)	4q21;5p15	Habitual miscarriage	Li et al., 2012 [23]
25	t(4;5)	4q21;5p15	Recurrent spontaneous abortion	Zhang M et al., 2015 [44]
26	t(4;5)	4q21;5q11.2	Severe oligoasthenoteratozoospermia	Peschka et al., 1999 [27]
27	t(4;5)	4q22;5q35	2 fetal losses	Adamoli et al., 1986 [37]
28	t(4;5)	4q25;5p15.2	4 abortions	Ghazaey et al., 2015 [45]
29	t(4;5)	4q31;5p15	Recurrent spontaneous abortions	Zhang et al., 2011 [46]
30	t(4;5)	4q31;5q13	normozoospermia	Huang et al., 2007 [47]
31	t(4;5)	4q32;5q14	Oligoasthenoteratozoospermia	Dohle et al., 2002 [48]
32	t(4;5)	4q32;5q14	Miscarriage	Dul et al., 2012 [49]
33	t(4;5)	4q35;5p15	Recurrent miscarriages	Dutta et al., 2011 [50]
34	t(5;6)	5p15.3;6q13	recurrent abortion	Kiss et al., 2009 [51]
35	t(5;6)	5p13.3;6q27	Recurrent spontaneous abortion	Gada Saxena et al., 2012 [33]
36	t(5;6)	5q21;6q33	Recurrent fetal wastage	Fryns et al., 1998 [34]
37	t(5;6)	5q33.1;6p11.2	Miscarriage	Sugiura-Ogasawara et al., 2008 [38]
38	t(5;6)	5q35;6p21.3	PGD	Ko et al., 2010 [52]
39	t(5;7)	5p15.2;7p14	Recurrent spontaneous abortion	Gada Saxena et al., 2012 [33]
40	t(5;7)	5p13;7p15	Recurrent pregnancy loss	Kochhar et al., 2013 [22]
41	t(5;7)	5p13;7p15	Spontaneous abortion	Stephenson et al., 2006 [53]
42	t(5;7)	5p11;7q11	8 abortions	Al-Hussain et al., 2000 [54]
43	t(5;7)	5q13;7p15.1	2 miscarriages	Estop et al., 1995 [55]
44	t(5;7)	5q21;7q32	Normozoospermia	Cifuentes et al., 1999 [56]
45	t(5;7)	5q33;7q22	Miscarriage and PGD	Kyu Lim et al., 2004 [30]
46	t(5;8)	5p14;8q22	Asthenozoospermia	Godo et al., 2013 [7]
47	t(5;8)	5q22;8q24.1	Oligoasthenoteratozoospermia	Meschede et al., 1998 [57]
48	t(5;8)	5q22.1;8q23.2	PGD	Ko et al., 2010 [52]
49	t(5;8)	5q23.1;8p23.2	4 miscarriage,1 term delivery	Ikuma et al., 2015 [4]
50	t(5;8)	5q33.3;8q11.21	Recurrent miscarriage	Pundir et al., 2016 [43]
51	t(5;8)	5q33;8q13	Normozoospermia	Blanco et al., 1998 [58]
52	t(5;8)	5q33;8q13	Normozoospermia	Estop et al., 2000 [59]
53	t(5;8)	5q33;8q13	Normozoospermia	Godo et al., 2013 [7]
54	t(5;8)	5q33;8q13	Normozoospermia	Anton et al., 2008 [60]
55	t(5;8)	5q35.1;8p11.2	Astenozoospermia	Anton et al., 2008 [60]
56	t(5;8)	5q35.3;8q22.1	Recurrent fetal wastage	Fryns et al., 1998 [34]
57	t(5;9)	5p15.1;9q22.1	Normospermic, Primary infertility	Vozdova et al., 2013 [11]
58	t(5;9)	5p13;9q22	PGD	Zhang et al., 2014 [61]
59	t(5;9)	5q10;9q10	Recurrent spontaneous abortions	Rouen et al., 2017 [62]
60	t(5;9)	5q21;9q34	2 fetal losses	Adamoli et al., 1986 [37]
61	t(5;9)	5q23.2;9q22.3	Spontaneous abortion	Stephenson et al., 2006 [53]
65	t(5;9)	5q23.3;9p24	Recurrent miscarriage	Iyer et al., 2007 [63]
63	t(5;10)	5p13.3;10p12.2	PGD	Ko et al., 2010 [52]
64	t(5;10)	5q22;10q11.2	PGD	Ko et al., 2010 [52]
65	t(5;10)	5q22;10q22	Miscarriage	Sugiura-Ogasawara et al., 2008 [38]
66	t(5;10)	5q34;10p12.1	Recurrent spontaneous abortions	Rouen et al., 2017 [62]
67	t(5;10)	5q35;10q22	Spontaneous abortions	Bourrouillou et al., 1986 [64]
68	t(5;10)	5q35;10q24	Recurrent miscarriage	Goud et al., 2009 [31]
69	t(5;11)	5p14;11p15	Normozoospermia	Zhang HG et al., 2015 [5]
70	t(5;12)	5p15.1;12p12.2	Spontaneous abortion	Stephenson et al., 2006 [53]
71	t(5;12)	5p15.1;12q21	Infertility	Ravel et al., 2006 [65]
72	t(5;12)	5p14;12q15	Recurrent spontaneous abortion	Gada Saxena et al., 2012 [33]
73	t(5;12)	5q11;12p13	10 abortions	Al-Hussain et al., 2000 [54]
74	t(5;12)	5q13;12q13	Recurrent spontaneous abortions	Rouen et al., 2017 [62]
75	t(5;12)	5q35.1;12q24.1	Repeated miscarriage	Goddijn et al., 2004 [66]
76	t(5;13)	5p13;13q34	Neonatal death	Zhang et al., 2006 [67]
77	t(5;13)	5q11;13q33	3 spontaneous abortions	Pellestor et al., 1989 [68]
78	t(5;13)	5q13;13q12	Normozoospermia	Zhang HG et al., 2015 [5]
79	t(5;13)	5q15;13p12	Oligozoospermia	Matsuda et al., 1992 [69]
80	t(5;13)	5q21;13q12.1	2 miscarriage, no conception	Ikuma et al., 2015 [4]
81	t(5;13)	5q33;13q12	Infertility	Mikelsaar et al., 2012 [20]
82	t(5;13)	5q34;13q33	Recurrent miscarriage	Iyer et al., 2007 [63]
83	t(5;14)	5p13;14q11.2	PGD	Zhang et al., 2014 [61]
84	t(5;14)	5p13;14q23	Spontaneous abortions	Bourrouillou et al., 1986 [64]
85	t(5;14)	5q11.2;14q32.1	Spontaneous abortion	Stephenson et al., 2006 [53]
86	t(5;15)	5p15.2;15q21.1	PGD	Ko et al., 2010 [52]
87	t(5;15)	5p13.3;15q15.3	PGD	Ko et al., 2010 [52]
88	t(5;15)	5q35;15q22	Pregnancy of PGD	Escudero et al., 2003 [70]
89	t(5;15)	5q35;15q26.2	Abnormal semen, 2 IVF-ET	Vozdova et al., 2013 [11]
90	t(5;16)	5q13; 16p13.1	Normozoospermia	Haapaniemi Kouru et al., 2017 [71]
91	t(5;16)	5q33;16p13	Recurrent pregnancy loss	Kochhar et al., 2013 [22]
92	t(5;16)	5q33.3;16p13.3	Recurrent miscarriages	Dutta et al., 2011 [50]
93	t(5;17)	5q13.2;17q21.2	infertility	Mau et al., 1997 [72]
94	t(5;17)	5q31;17p13	Normozoospermia	Anton et al., 2008 [60]
95	t(5;17)	5q33.1;17q25.3	Repeated miscarriage	Goddijn et al., 2004 [66]
96	t(5;18)	5p15;18q11.2	Spontaneous abortion	Zhang HG et al., 2015 [5]
97	t(5;18)	5p15;18q21	Malformed children	Balkan et al., 1983 [73]
98	t(5;18)	5q15;18q22	Spontaneous abortions	Soh et al., 1984 [74]
99	t(5;18)	5q15;18q23	2 fetal loss	Smith et al., 1990 [75]
100	t(5;18)	5q31.1;18q21.1	PGD	Ko et al., 2010 [52]
101	t(5;19)	5q15;19p12	Normospermic, A boy 46,XY,t(5;19)pat	Vozdova et al., 2013 [11]
102	t(5;20)	5q22;20p13	Asthenozoospermia, Habitual abortions	Vegetti et al., 2000 [32]
103	t(5;20)	5q13;20q12	Normozoospermia	Zhang HG et al., 2015 [5]
104	t(5;20)	5q22;20p12	Recurrent fetal wastage	Fryns et al., 1998 [34]
105	t(5;20)	5q31;20p13	Azoospermia	Poli et al., 2016 [18]
106	t(X;5)	Xp22.1;5p11	Azoospermia	Peschka et al., 1999 [27]

The most common breakpoint, at 5p15, was observed in 11 patients, followed by 5q13 (*N* = 10). Breakpoints at 5p14, 5p11, 5q13, 5q14, 5q15, 5q22, 5q31, 5q35 and 5q35.1 were found with cases of both pregestational and gestational infertility. All breakpoints were associated with gestational infertility (Table 3).

**Table 3:** Incidence of breakpoints on chromosome 5

Breakpoints	Number of patients with pregestational infertility	Number of patients with gestational infertility
p15.3		1
p15.2		3
p15.1		4
p15		11
p14	1	5
p13.3		3
p13		9
p12		2
p11	1	2
q10		1
q11		3
q11.2		2
q12		1
q13	1	9
q13.1		1
q13.2		1
q14	1	1
q15	1	4
q21		4
q22	2	3
q22.1		1
q23.1		1
q23.2		1
q23.3		1
q31	2	3
q31.1		1
q33		8
q33.1		2
q33.2		1
q33.3		3
q34		2
q35	1	6
q35.1	1	2
q35.3		2

## Discussion

In clinical practice, male infertility can be broadly divided into two types of reproductive failure: pregestational and gestational infertility [16]. In the present study, nine of our cases were identified as carriers of chromosome 5 translocations, and we also reviewed 106 cases of chromosome 5 translocation reported in the literature. The breakpoints that we identified on chromosome 5 were found to be associated with pregestational or gestational infertility. One case was associated with pregestational infertility and eight cases were related to gestational infertility. Mikelsaar et al. [17] and Venkateshwari et al. [18] reported that the breakpoints at 5q33 and 5q35 in male carriers were associated with infertility. Kim et al. [19] reported that the breakpoint at 5p13 could interfere with spermatogenesis, and that breakpoints at 5q15, 5q21.2, 5q22 and 5q32 were related to recurrent abortion. To study the relationship of these breakpoints on chromosome 5 with male infertility, we analyzed recent published literature and revealed clinical features in carriers of chromosome 5 translocations. The karyotype results and clinical findings at chromosome 5 are summarized in Table 2. A common clinical feature associated with the breakpoints at 5p13, 5q33 and 5q35 was recurrent miscarriage, which was not consistent with the above reports.

Male infertility affects about 50% of couples unable to achieve pregnancy [20]. Chromosomal abnormalities are closely related to male infertility [21], and cytogenetic detection can provide valuable information for genetic counseling of infertile males [22]. Previous reports have shown that infertile men have an 8–10-fold higher prevalence of chromosomal abnormalities than fertile men [19]. Chromosomal translocation alters the complex and vital process of spermatogenesis, and leads to male infertility [20]. Chromosome 5 translocation has often been associated with male infertility or recurrent pregnancy loss [17, 18, 23].

Table 3 shows that all breakpoints were associated with gestational infertility. These cases indicated that such breakpoints were not responsible for pregestational infertility, so another breakpoint of translocation must be responsible in these individuals. For instance, two individuals with the breakpoint 5q22 were associated with pregestational infertility, and exhibited clinical features of oligoasthenoteratozoospermia and asthenozoospermia (case 47 and 102, respectively, Table 2). The corresponding breakpoints of translocation in case 47 and 102 were 8q24.1 and 20p13, respectively. Kott et al. [24] reported that the primary ciliary dyskinesia-19 (*CILD19*) gene (OMIM: 614,935), mapped to chromosome 8q24, was associated with asthenospermia in infertile males. Previous studies have shown that the sperm flagellar protein 1 (*SPEF1*) gene (OMIM: 610,674) located on chromosome 20p13 was be associated with curvature of microtubule bundles and the axoneme of sperm flagella [25]. Previous studies suggested that disruptions of *CAMK4* located on chromosome 5q22.1, *SPINK13* located on chromosome5q32 and the testis-specific serine/threonine kinase (*TSSK1B*) gene mapped to chromosome 5q22.2 were associated with loss of sperm function and human male infertility [8, 9, 26]. In addition, the most common breakpoint, mapped to 5p15, was associated with gestational infertility. Other breakpoints were also identified as being associated with gestational infertility. For those affected by these breakpoints, natural conception remained possible with the potential to have normal children. For example, Ikuma et al. [4] reported that the live birth rate with natural conception for translocation carriers was 65%–83% cumulatively. However, natural conception has a greater risk. The number of chromosomal unbalanced gametes is large, leading to repetitive pregnancy loss, and may have repercussions on the fertility of translocation carriers. For these carriers, informed choice should be provided during genetic counseling.

The major limitation of our present study was the relatively small number of carriers of chromosome 5 translocations. Furthermore, we did not investigate the specific molecular effects of the translocations by molecular-cytogenetic methods.

## Conclusions

In the present study, 115 carriers of chromosome 5 translocations were reviewed. The most common translocation and breakpoint were t(4;5) and 5p15, respectively. All breakpoints at chromosome 5 were associated with gestational infertility. In genetic counseling, physicians should consider chromosome 5 and its breakpoints. Carriers of chromosome 5 translocations maybe choose to continue with natural conception or use available assisted reproductive technologies, such as preimplantation genetic diagnosis.

## Abbreviations

AZF: Azoospermia factor
CAMK4: Ca^2+^/calmodulin-dependent protein kinase IV
CILD19: Ciliary dyskinesia-19
ISCN: International System for Human Cytogenetic Nomenclature
PGD: Preimplantation genetic diagnosis
SPEF1: Sperm flagellar protein 1
Spink13: Serine protease inhibitor, Kazal-type 13
TSSK1B: Testis-specific serine/threonine kinase
